# Generational synaptic functions of GABA_A_ receptor β3 subunit deteriorations in an animal model of social deficit

**DOI:** 10.1186/s12929-022-00835-w

**Published:** 2022-07-11

**Authors:** Ming-Chia Chu, Han-Fang Wu, Chi-Wei Lee, Yueh-Jung Chung, Hsiang Chi, Po See Chen, Hui-Ching Lin

**Affiliations:** 1 Department and Institute of Physiology, School of Medicine, National Yang Ming Chiao Tung University, Tainan, 112 Taiwan; 2 Ph.D. Program for Neural Regenerative Medicine, College of Medical Science and Technology, Taipei Medical University and National Health Research Institutes, Taipei, 110 Taiwan; 3 Department of Psychiatry, Institute of Behavioral Medicine, College of Medicine and Hospital, National Cheng Kung University Hospital, National Cheng Kung University, No. 138, Sheng-Li Road, Tainan, 704 Taiwan; 4 Institute of Behavioral Medicine, College of Medicine, National Cheng Kung University, Tainan, 704 Taiwan; 5 Brain Research Center, National Yang Ming Chiao Tung University, Taipei, 112 Taiwan

**Keywords:** GABA_A_R, Excitatory/inhibitory imbalance, Gephyrin, Generational effect, Valproate, Autism spectrum disorder

## Abstract

**Background:**

Disruption of normal brain development is implicated in numerous psychiatric disorders with neurodevelopmental origins, including autism spectrum disorder (ASD). Widespread abnormalities in brain structure and functions caused by dysregulations of neurodevelopmental processes has been recently shown to exert adverse effects across generations. An imbalance between excitatory/inhibitory (E/I) transmission is the putative hypothesis of ASD pathogenesis, supporting by the specific implications of inhibitory γ-aminobutyric acid (GABA)ergic system in autistic individuals and animal models of ASD. However, the contribution of GABAergic system in the neuropathophysiology across generations of ASD is still unknown. Here, we uncover profound alterations in the expression and function of GABA_A_ receptors (GABA_A_Rs) in the amygdala across generations of the VPA-induced animal model of ASD.

**Methods:**

The F2 generation was produced by mating an F1 VPA-induced male offspring with naïve females after a single injection of VPA on embryonic day (E12.5) in F0. Autism-like behaviors were assessed by animal behavior tests. Expression and functional properties of GABA_A_Rs and related proteins were examined by using western blotting and electrophysiological techniques.

**Results:**

Social deficit, repetitive behavior, and emotional comorbidities were demonstrated across two generations of the VPA-induced offspring. Decreased synaptic GABA_A_R and gephyrin levels, and inhibitory transmission were found in the amygdala from two generations of the VPA-induced offspring with greater reductions in the F2 generation. Weaker association of gephyrin with GABA_A_R was shown in the F2 generation than the F1 generation. Moreover, dysregulated NMDA-induced enhancements of gephyrin and GABA_A_R at the synapse in the VPA-induced offspring was worsened in the F2 generation than the F1 generation. Elevated glutamatergic modifications were additionally shown across generations of the VPA-induced offspring without generation difference.

**Conclusions:**

Taken together, these findings revealed the E/I synaptic abnormalities in the amygdala from two generations of the VPA-induced offspring with GABAergic deteriorations in the F2 generation, suggesting a potential therapeutic role of the GABAergic system to generational pathophysiology of ASD.

**Supplementary Information:**

The online version contains supplementary material available at 10.1186/s12929-022-00835-w.

## Background

The prevalence of neurodevelopmental disorders has been dramatically risen worldwide during the last few decades [1, 2]. Early life disturbance in the brain is implicated in numerous psychiatric disorders with neurodevelopmental origins, including autism spectrum disorder (ASD). Recent evidence has strengthened the relationship between parental life experiences and offspring physiology [3, 4]. Environmental perturbations, such as parental stress, malnutrition, infection, or toxicants, increase the offspring susceptibility of ASD and other neurodevelopmental disorders [5–7]. Moreover, widespread abnormalities in brain structure and functions caused by disruptions of neurodevelopmental processes has been recently shown to enhance the incidence of disease and exert adverse effects across generations in both clinical and animal studies [7–12]. Several brain structures are highly influenced by parental mental health in subsequent generations of offspring, including the amygdala and hippocampus [13–15]. This includes the significant association between the amygdala and socio-emotional problems in individuals with ASD that has long been identified [16–19]. The altered excitatory and inhibitory synaptic functions in the amygdala are regarded as a leading cause of the autism phenotype [20, 21]. Therefore, uncovering the key hallmarks within a specific brain area underlying the cross-generational impact of ASD has become a matter of great urgency.

Excitatory/inhibitory (E/I) imbalance within the brain is one of the pathophysiological theories of ASD [22]. In line with this, both post-mortem and animal studies have observed abnormal dendritic spine morphology [23, 24] and altered levels of glutamate- and γ-aminobutyric acid (GABA)-related proteins in the brain tissue of patients or animals with ASD [25–29]. Recent evidence assessed by next-generation mRNA sequencing has revealed several transcriptional alterations in the glutamatergic signaling implicated in schizophrenia, bipolar disorder and ASD in the F1 and F2 generations of the offspring derived from immune-challenged ancestors [30]. Moreover, the transgenerational replicability of abnormal glutamatergic protein expressions has been demonstrated in an ASD model induced by prenatal VPA exposure [31]. Another key factor in the E/I balance is the essential role of the γ-aminobutyric acid (GABA)ergic system, which has long been documented in ASD [32]. Specifically, the β3 subunits of GABA_A_R are vital for the proper functioning of various brain regions during neurodevelopment. Mutations or duplications of chromosome 15q11-13, a complex locus containing genes for the β3 subunits of GABA_A_R, is the most common chromosomal anomaly in cases of ASD [33–35]. Moreover, disruptions in the GABA_A_R β3 subunit have been emphasized for their direct contribution to the pathophysiology of ASD in animal models [36–38]. Collectively, these evidences increase the possibility that dysfunctions of the GABAergic system, especially the GABA_A_R β3 subunit, is a pathological hallmark of various neurodevelopmental diseases; therefore, elucidating its role in the cross-generational effects of ASD pathogenesis is necessary.

Accordingly, the present study aimed to investigate whether E/I alterations, specifically in GABAergic system, contribute to the cross-generational effects on the pathogenesis of ASD by using VPA-induced animal model of ASD. We generated a F2 generation of the VPA-induced offspring by mating the F1 VPA-induced male offspring with naïve females after a single injection of VPA on an embryonic day (E12.5) in F0. We show that both two generations of the VPA-induced offspring performed autistic behavioral phenotypes with synaptic aberrations in both excitatory and inhibitory systems in the amygdala. Intriguingly, greater reductions in the GABAergic tone in the F2 generation of the VPA-induced offspring were observed compared with the F1 generation in this animal model of ASD.

## Methods

### Animals

All experimental procedures complied with the National Institute of Health Guide for the Care and Use of Laboratory Animals (USA) and approved by the Institutional Animal Care and Use Committee at National Yang Ming Chiao Tung University with a project number 1091206n. Animals were housed in a controlled temperature (24 ± 1 °C) and humidity (50 ± 5%) under a 12 h light/dark schedule with food and water available ad libitum. Eight-to ten-week-old male and female Sprague Dawley (SD) rats were mated in pairs. Female rats were controlled every morning, and the presence of a vaginal plug was considered as embryonic day (E0.5). Single intraperitoneal administration of 500 mg/kg sodium valproate (NaVPA) was delivered to pregnant rats on an E12.5 for producing F1 generation VPA-induced offspring, whereas the control group received sterile saline (500 mg/kg) as vehicle. F2 generation of VPA-induced offspring were generated by mating the male VPA-induced F1 offspring with naïve females (Additional file 1: Fig. S1a). Pregnant rats were housed individually and allowed to raise their own litters. Litter size was kept at 8–12 pups per dams by culling at postnatal day 3 (PND3) with even distribution between male and female. After weaning on PND21, pups from same litter were group housed in same-sex (3–4 pups/cage) and subjected to rest of the behavioral assays during the light cycle from PND28 to PND32. Western blotting and electrophysiological assessments were performed immediately after behavioral tests (Additional file 1: Fig. S1b). Three to four male offspring were randomly selected from each litter, and overall three or four litters per group were evaluated in present study to avoid litter effects.

### Drug and antibodies

For prenatal VPA exposure, NaVPA was purchased from Sigma-Aldrich (St. Louis, MO), which was dissolved in 0.9% saline to obtain a concentration of 150 mg/ml at pH 7.3. For GABA_A_R insertion, *N*-methyl-d-aspartate (NMDA) was purchased from Tocris (Minneapolis, MN), which was dissolved in artificial cerebrospinal fluid (aCSF) to obtain a concentration of 20 ${\upmu }$M. For western blotting, the following antibodies of GluN2A (1:5000; Genetex; GTX63442), GluN2B (1:5000; Abcam; ab65783), GluA1 (1:5000; Abcam; ab109450), GluA2 (1:5000; Millipore; MAB397), gephyrin (1:5000; Alomone; AIP-005), GABA_A_R β3 subunit (1:5000; Abcam; ab98968), and anti-${\upbeta }$-actin antibody (1:10,000; Abcam; ab6276) were used.

### Behavioral testing

All behavioral procedures were recorded using a digital video camera. Behavioral traces of rat movement during the experiments were recorded through Smart software (version 3.0; Panlab, S.L.U., Spain). The behavioral testing was performed and scored by researchers blind to the experimental conditions of the test rat.

#### Three-chamber social interaction test

The three-chamber social interaction test was performed based on the procedure described previously [39]. The social interaction testing apparatus was an open-topped Plexiglas box (30 × 60 × 30 cm) divided into three compartments. Among them, two identical, transparent boxes (28 × 13 × 21 cm) were respectively positioned on both side compartments. The test was composed of three phases with different stimuli placed in each of the plastic boxes. First, during the habituation phase (5 min), no stimulus was involved. During the sociability phase (5 min), a social stimulus (stranger rat 1, S1) was enclosed in one of the transparent box in a side compartments. During the social preference phase (5 min), the stranger rat 1 (familiar, F) and a novel social stimulus (stranger rat 2, S2) were placed in each of the plastic boxes, respectively. Within each phase, test rat was introduced to the center of the apparatus and allowed for free exploration. Animals used as strangers were rats of similar age, same sex, and similar weight as the test rat. The amount of time spent in each compartment was measured. The preference index for sociability was calculated as time spent in compartment (S1 – E)/total exploration time $\times$ 100%, where E denotes the empty plastic box. The preference index for social preference was measured as time spent in compartment (S2 – F)/total exploration time $\times$ 100%.

#### Marble-burying test

Standard rat cages were filled with fresh bedding to a depth of 4 cm and were embedded with 20 evenly distributed marbles (~ 1.5 cm diameter). Rats were placed in the cage for 20 min and were allowed to explore freely. Afterward, the number of marbles that were more than two-thirds buried was counted [40].

#### Open-field test

The open-field test was conducted in a novel square arena (45 × 45 cm) surrounded by walls (45 cm high) made of black Plexiglas. At the start of each trial, rat was placed in the center of the apparatus and allowed to freely ambulate the apparatus for 5 min. The total distance moved and the percentage of time spent in the central 25% of the apparatus during the experiment were measured.

#### Elevated plus maze test

The elevated plus maze test was made of black Plexiglas and elevated at a height of 31 cm above the floor level. The plus maze contains two open arms (112 × 112 cm) and two enclosed arms (112 × 112 × 31 cm) radiating out from a central platform (10 × 10 cm). Rats were placed in the center of the maze and their movements were monitored over a 10-min period. Total time spent in the open arms of the maze was calculated and presented as a percentage of the test duration.

#### Forced swim test

During the forced swim test, rats were individually subjected to a 5-min swim session in a transparent Plexiglas cylinder, 60 cm high and 21 cm in diameter, filled with water (25 $\pm$ 1 °C). Immobility was defined as the absence of active, escape-oriented behaviors, with only slight movements to keep the head above water [40].

### Electrophysiology

Amygdala slices were prepared as previously described [41]. Rats were decapitated, and brains were rapidly removed and placed in ice-cold high sucrose slicing solution consisted of the following (in mM): sucrose 75, NaCl 87, KCl 2.5, CaCl_2_ 0.5, MgCl_2_ 4, NaHCO_3_ 23, NaH_2_PO_4_ 1.25, and glucose 25 at pH 7.4, and equilibrated with 95% O_2_–5% CO_2_. Brain slices (400 $\upmu$m) were then prepared through a vibrating microtome (DTK-1000; Dosaka, Kyoto, Japan) and further transferred to a holding chamber of normal aCSF, saturated with 95% O_2_ and 5% CO_2_ and containing the following (in mM): NaCl 130, KCl 2.5, CaCl_2_ 1.2, MgCl_2_ 2.4, NaHCO_3_ 23, NaH_2_PO_4_ 1.2, and glucose 11 at pH 7.4, 30–32 °C.

During recording, an individual slice was placed in a recording chamber on an upright microscope stage (Olympus BX51W1, Tokyo, Japan) and constantly superfused at 2–5 ml/min with oxygenated aCSF at 30–32 °C. Pyramidal neurons in the basolateral amygdala (BLA) subregion of the amygdala were visualized using a NIR-sensitive CCD camera (acA2040-90 μm; Baseler). A concentric bipolar stimulating electrode (FHC, Boedoinham, ME, USA) placed outside of the BLA to stimulate the external capsule (EC) fibers from the cortex.

Whole-cell patch clamp recordings were obtained from visually identified pyramidal neurons in the BLA via a capillary glass microelectrode (4–5 MΩ). Electrode internal was composed of the following (in mM): K or Cs-gluconate 140, KCl or CsCl 10, EGTA 1, phosphocreatine 10, Mg-ATP 4, Na-GTP 0.3, and HEPES 10 at pH 7.3, 280 mOsm. For recording neuronal properties and action potentials, K-based internal solution was used, whereas for recording ${\upalpha }$-amino-3-hydroxy-5-methyl-4-isoxazolepropionic acid receptors (AMPAR)/NMDA receptor (NMDAR) ratio and inhibitory post-synaptic currents (IPSCs), Cs-based internal solution was used. All recordings were monitored and digitized through MultiClamp 700B and Digidata1322A, respectively. Excitatory post-synaptic currents (EPSCs) were performed in the presence of tetrodotoxin (1 ${\upmu }$M) and picrotoxin (10 ${\upmu }$M) in the recording aCSF. NMDAR-mediated EPSC was determined as amplitude at 50 ms after peak EPSC amplitude holding at 40 mV, whereas AMPAR-mediated EPSC was evoked as the cell voltage-clamped at – 70 mV. The AMPAR/NMDAR ratio was estimated by calculating AMPAR-mediated EPSC amplitude divided by NMDAR-mediated EPSC amplitude. GABA_A_R-mediated miniature IPSCs were performed with the cell held at – 70 mV and the presence of 6-cyano-7-nitroquinoxaline-2,3-dione (CNQX; 10 $\upmu$M) and d-2-amino-5-phosphonovaleric acid (d-APV; 50 $\upmu$M) in the recording aCSF. The input–output curve and the reversal potential of evoked IPSCs was measured by averaging 10 recorded responses for each tested stimulation intensity and each holding potential, respectively. As described in the previous study [42], for the potentiation of GABAergic synapses, NMDA (3 min, 20 ${\upmu }$M) was briefly applied.

### Western blotting and immunoprecipitation

#### Whole-cell lysate preparation

The BLA subregion of the amygdala was punched from brain slices, and the tissue was then homogenized in iced-cold lysis buffer with proteinase inhibitors and phosphatase inhibitors. The prepared homogenate was centrifuged at 12,000$\times$*g* for 20 min, and the supernatant was used for western blotting analysis.

#### Synaptoneurosome preparation

The BLA subregion of the amygdala stimulated with NMDA or with sham solution were punched from brain slices and synaptoneurosomal fraction was prepared according to the procedure of described previously [41, 43]. The tissue was homogenized in iced-cold lysis buffer with proteinase inhibitors and phosphatase inhibitors. The mixture was loaded into a 1-ml tuberculin syringe attached to a 13 mm diameter syringe filter holder (Millipore). After filtration, the mixture was forced to pass over a three-layer nylon (Tetko, 100-$\upmu$m pore diameter) rinsed with lysis buffer. The filtrate was loaded into another tuberculin syringe and forced through a pre-wetted nitrocellulose filter (5 $\upmu$m, Millipore). The filtered homogenate was then centrifuged at 1000$\times$*g* for 10 min, and the pellet was resuspended in lysis buffer for western blotting analysis.

#### Immunoprecipitation

The synaptoneurosomes were immunoprecipitated with anti-GABA_A_R β3 subunit (2 ${\upmu }$g) antibodies, or IgG in iced-cold lysis buffer overnight. The antibody-bound complex were incubated with protein G-coupled agarose beads at 4 °C for 1 h. The agarose beads were pelleted by centrifugation. After wash and elution, the immunoprecipitates were detected with anti-gephyrin antibody by western blotting analysis.

#### Western blotting analysis

For analysis of synaptoneurosomes, whole-cell lysates and prepared immunoprecipitates, a fixed amount of protein was subjected to western blotting. Protein concentration of prepared sample was determined by the Bio-Rad protein assay. Equal volume of 5× sample buffer (10% SDS, 250mM Tris-HCl, pH 6.8, 5% $\beta$-mercaptoethanol, 50% glycerol and 0.5% bromophenol blue) were added to samples, and boiled for 10 min. After that, protein extracts separated on 7.5% SDS-PAGE and transferred to PVDF (Immobilon P membrane, Millipore). The membranes were blocked with 5% non-fat milk for 1 h at room temperature and incubated overnight with primary antibodies at 4 °C. Immune complexes were detected by using appropriate HRP-conjugated secondary antibodies along with ECL Plus detection reagent (PerkinElmer, Boston, MA, USA). Signals were acquired via *x*-ray films (Fujifilm Super RX). Signal intensities of proteins were determined by Image J software and were normalized to internal control for each individual sample.

### Statistical analysis

All experiments are performed in a blinded manner. Statistical analyses were performed with GraphPad Prism 6. All values were expressed as the mean ± SEM. Experiments were analyzed statistically using one-way ANOVA with Bonferroni post hoc tests or two-way repeated measure ANOVA (rmANOVA) with Bonferroni post hoc tests. Probability value (*p*) $\le$ 0.05 was considered statistically significant.

## Results

### Autism-like behaviors across two generations of the VPA-induced offspring

**Fig. 1:**
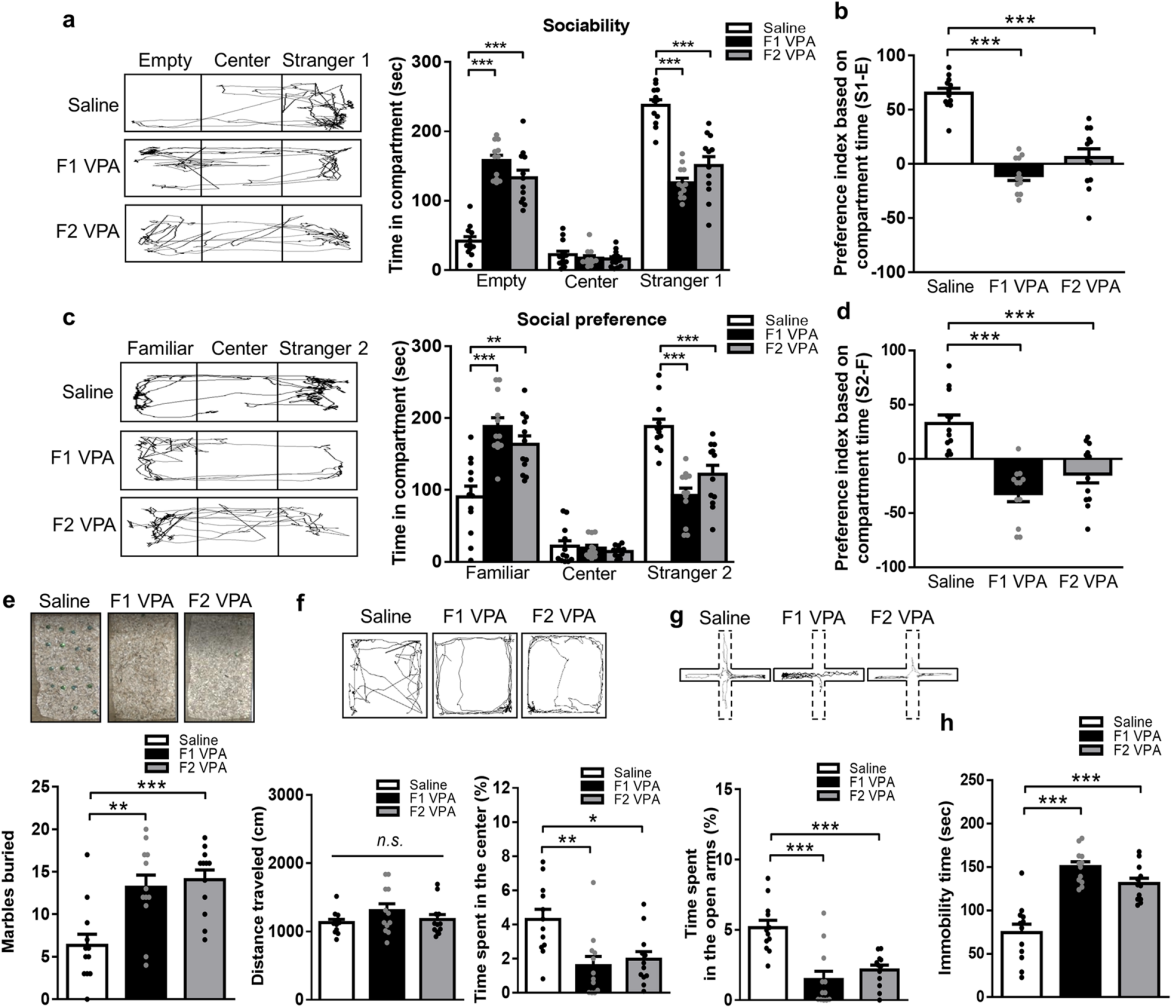
Autism-like behaviors across two generations of the VPA-induced offspring. **a** Representative traces and time spent in three compartments calculated during the phase 2 of the three-chamber social test. **b** The preference index for sociability (%) calculated during the phase 2 of the three-chamber social test. **c** Representative traces and time spent in three compartments calculated during the phase 3 of the three-chamber social test. **d** The preference index for social preference (%) calculated during the phase 3 of the three-chamber social test. **e** Representative traces and marble buried recorded during the marble burying test. **f** Representative traces, total distance traveled, and percentage of time spent in the center by the animals during the open field test. **g** Representative traces and time spent in the open arms measured during the elevated plus maze test. **h** Immobility time calculated during the forced swim test. *n* = 12 rats from three or four litters for each condition. Data are presented as mean ± SEM; **p < 0.01, ***p < 0.001 vs. saline-exposed offspring; one-way ANOVA with Bonferroni post-hoc

First, the three-chamber social interaction test was performed. In the sociability phase, less exploration time with stranger rat 1 (*F*_(2,33)_ = 37.70, *p* < 0.0001; Fig. 1a) and decreased preference index (*F*_(2,33)_ = 45.83, *p* < 0.0001; Fig. 1b) were observed in two generations of the VPA-induced offspring relative to the saline-exposed offspring. In the social preference phase, less exploration time with the novel stranger rat (*F*_(2,33)_ = 19.77, *p* < 0.0001; Fig. 1c) and diminished preference index (*F*_(2,33)_ = 18.34, *p* < 0.0001; Fig. 1d) were found in two generations of the VPA-induced offspring compared with the saline-exposed offspring. In the marble burying test, increased marbles buried were revealed in two generations of the VPA-induced offspring relative to the saline-exposed offspring (*F*_(2,33)_ = 10.45, *p* = 0.0003; Fig. 1e). In the open field test, no significant change in the total distance traveled among the three groups was detected (*F*_(2,33)_ = 1.475, *p* = 0.2434), while reduced time spent in the center area were found in two generations of the VPA-induced offspring compared with the saline-exposed offspring (*F*_(2,33)_ = 7.592, *p* = 0.0019; Fig. 1f). In the elevated plus-maze test, significantly decreased time spent in the open arms were shown in two generations of the VPA-induced offspring compared with the saline-exposed offspring (*F*_(2,33)_ = 15.72, *p* < 0.0001; Fig. 1g). In the forced swim test, enhanced immobility time was found in two generations of the VPA-induced offspring compared with the saline-exposed offspring (*F*_(2,33)_ = 28.54, *p* < 0.0001; Fig. 1h). Collectively, these data support the hypothesis that maternal VPA exposure results in autism-relevant behaviors across two generations of the VPA-induced offspring.

### Decreased synaptic GABA_A_R levels and inhibitory transmission in two generations of the VPA-induced offspring with further reductions in the F2 generation

**Fig. 2:**
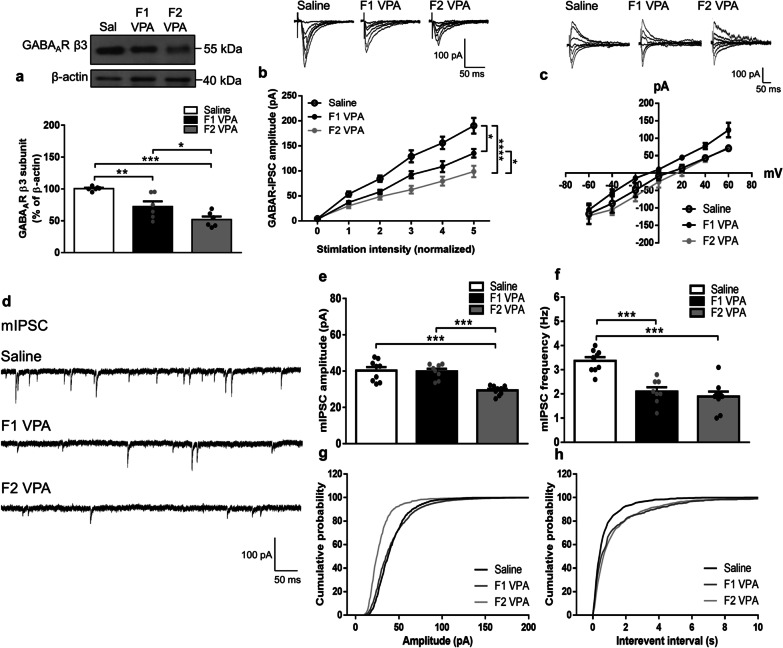
Synaptic GABA_A_R levels and inhibitory transmission in two generations of the VPA-induced offspring. **a** Representative western blot profile and the protein levels of GABA_A_R ${\upbeta }$3 subunit in the synaptoneurosomes from the amygdala. *n* = 6 rats from three or four litters for each condition. **b** Top, Representative IPSC traces at different stimuli. Scale bars, 100 pA, 50 ms. Bottom, input–output curves of IPSC in response to a series of increasing stimulus intensities. *n* = 9–10 cells from 4–5 rats from three or four litters for each condition. **c** Top, Representative IPSC traces at different holding potentials. Scale bars, 100 pA, 50 ms. Bottom, reversal potential of IPSCs in response to various holding potential ranging from − 60 to 60 mV. *n* = 8–10 cells from 4–5 rats from three or four litters for each condition. **d** Representative mIPSCs traces from the BLA pyramidal neurons. Scale bars, 100 pA, 50 ms. **e**, **f** Bar graphs show average mIPSC amplitudes (**e**) and frequency (**f**) measured from the BLA pyramidal neurons. **g**,** h** Cumulative probability of mIPSC amplitudes (**g**) and frequency (**h**) measured from the BLA pyramidal neurons. *n* = 8–9 cells from 4–5 rats from three or four litters for each condition. Data are presented as mean ± SEM; *p < 0.05, **p < 0.01, ***p < 0.001 vs. indicated control; one-way ANOVA or two-way rmANOVA with Bonferroni post-hoc

To address the contribution of GABAergic system to the heritable effects of maternal VPA exposure, GABA_A_R expression and function were assessed. As shown in Fig. 2a, both generations of the VPA-induced offspring displayed significant reductions in the synaptic protein levels of the GABA_A_R β3 subunit in the amygdala; moreover, the reduction level was significantly greater in the F2 than the F1 generation (*F*_(2,15)_ = 19.99, *p* < 0.0001). Consistently, the input–output relationships were decreased in both generations of the VPA-induced offspring; furthermore, the level was significantly lower in the F2 compared to the F1 generation of the VPA-induced offspring (Saline vs. F1 VPA, interaction: *F*_(5,102)_ = 2.585, *p* = 0.0303; Saline vs. F2 VPA, interaction: *F*_(5,108)_ = 7.825, *p* < 0.0001; F1 VPA vs. F2 VPA, interaction: *F*_(5,102)_ = 2.345, *p* = 0.0465; Fig. 2b). No significant differences were found among the groups in the reversal potentials of IPSCs (interaction: *F*_(12,168)_ = 0.3392, *p* = 0.9808; Fig. 2c). The extent of mIPSC amplitude (*F*_(2,23)_ = 17.66, *p* < 0.0001; Fig. 2e, g) was significantly declined in the F2 generation of the VPA-induced offspring either compared with the saline-exposed offspring or the F1 generation offspring. As shown in Fig. 2f, h, both two generations of the VPA-induced offspring revealed significant reductions in the mIPSC frequency compared with the saline-exposed offspring (*F*_(2,23)_ = 19.89, *p* < 0.0001). Altogether, these data indicated diminished synaptic GABA_A_R expressions and inhibitory transmission in two generations of the VPA-induced offspring with greater reductions in the F2 generation.

### Loss of synaptic gephyrin levels in two generations of the VPA-induced offspring with a greater reduction in the F2 generation accompanied with weaker association of gephyrin with GABA_A_R

**Fig. 3:**
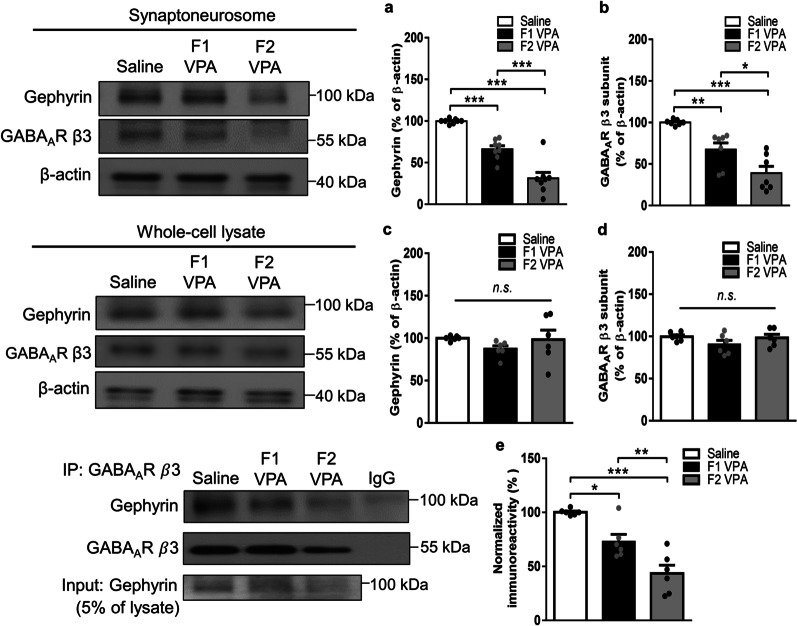
Synaptic gephyrin levels and gephyrin-GABA_A_R associations in two generations of the VPA-induced offspring. **a**,** b** Representative western blot profile and the protein levels of gephyrin (**a**) and GABA_A_R ${\upbeta }$3 subunit (**b**) in synaptoneurosomes from the amygdala. Gephyrin, *n* = 8 rats from three or four litters for each condition; GABA_A_R ${\upbeta }$3 subunit, *n* = 7 rats from three or four litters for each condition. **c**,** d** Representative western blot profile and the protein levels of gephyrin (**c**) and GABA_A_R ${\upbeta }$3 subunit (**d**) in whole-cell lysate from the amygdala. Gephyrin, *n* = 6 rats from three or four litters for each condition; GABA_A_R ${\upbeta }$3 subunit, *n* = 6 rats from three or four litters for each condition. (**e**) Representative western blot profile and the protein levels of co-immunoprecipitation of gephyrin and GABA_A_R ${\upbeta }$3 subunit in synaptoneurosomes from the amygdala. *n* = 6 rats from three or four litters for each condition. Data are presented as mean ± SEM; *p < 0.05, **p < 0.01, ***p < 0.001 vs. indicated control; one-way ANOVA with Bonferroni post-hoc

To identify the molecular mechanism underlying the deterioration of the GABAergic system across two generations of the VPA-induced offspring, we focused on the expression profiles of a scaffold protein, gephyrin, known to play important role in the clustering and stabilization of GABA_A_Rs at inhibitory postsynapses [44]. Western blot analyses of synaptoneurosomal tissue showed that the expression levels of gephyrin and GABA_A_R β3 subunit were significantly decreased in both of the F1 and F2 generations of the VPA-induced offspring compared to the saline-exposed offspring, and greater reduction levels were observed in the F2 than the F1 generation (Gephyrin: *F*_(2,21)_ = 52.49, *p* < 0.0001; GABA_A_R ${\upbeta }$3 subunit: *F*_(2,18)_ = 21.4, *p* < 0.0001; Fig. 3a, b). No significant difference was found in gephyrin and GABA_A_R β3 subunit levels among the three groups in the whole-cell fraction from the amygdala (Gephyrin: *F*_(2,15)_ = 1.022, *p* = 0.3837; GABA_A_R β3 subunit: *F*_(2,15)_ = 1.609, *p* = 0.2328; Fig. 3c, d). In immunoprecipitation experiments, the association of the GABA_A_R β3 subunit and gephyrin was significantly attenuated in both F1 and F2 generations of the VPA-induced offspring compared to the saline-exposed offspring, and greater loss were measured in the F2 than the F1 generation of the VPA-induced offspring (*F*_(2,15)_ = 23.01, *p* < 0.0001; Fig. 3e). In summary, these data indicated declined gephyrin and GABA_A_R levels, and their association in two generations of the VPA-induced offspring with further reductions in the F2 generation, suggesting that clustering of gephyrin and GABA_A_R at the synapses is dysregulated in both generations of the VPA-induced offspring with deterioration in the F2 generation.

### Defective NMDA-induced gephyrin and GABA_A_R enhancements in two generations of the VPA-induced offspring with difference between two generations

**Fig. 4:**
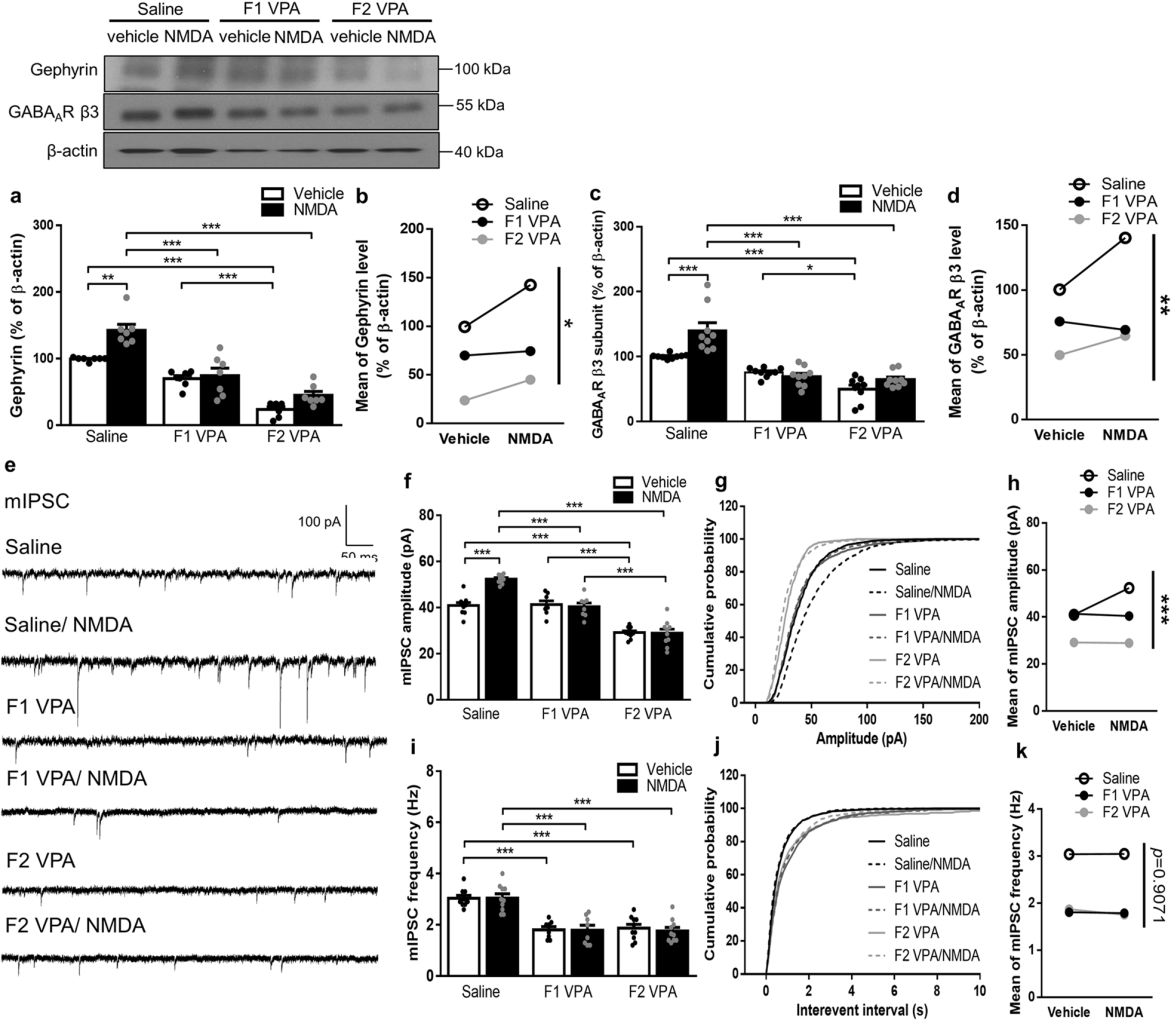
NMDA-induced gephyrin and GABA_A_R enhancements in two generations of the VPA-induced offspring. **a–d** Representative western blot profile, the protein levels (**a**,** c**), and the main effect plots of two-way ANOVA (**b**,** d**) of gephyrin and GABA_A_R ${\upbeta }$3 subunit in synaptoneurosomes from the amygdala. Gephyrin, *n* = 7 rats from three or four litters for each condition; GABA_A_R ${\upbeta }$3 subunit: *n* = 9 rats from three or four litters for each condition. **e** Representative mIPSCs traces from each group. Scale bars, 100 pA, 50 ms. **f**,** i** Bar graphs show average mIPSC amplitudes (**f**) and frequency (**i**) measured from the BLA pyramidal neurons. **g**,** j** Cumulative probability of mIPSC amplitudes (**g**) and frequency (**j**) measured from the BLA pyramidal neurons. **h**,** k** The main effect plots of two-way ANOVA of mIPSC amplitudes (**h**) and frequency (**k**) measured from the BLA pyramidal neurons. *n* = 8–10 cells from 4–5 rats from three or four litters for each condition. Data are presented as mean ± SEM; *p < 0.05, ***p < 0.001 vs. indicated control; one-way ANOVA or two-way rmANOVA with Bonferroni post-hoc

To further investigate the impact of lossing synaptic gephyrin and GABA_A_R on inhibitory synapses in two generations of the VPA-induced offspring, we evoked the potentiation of inhibitory synapses accompanied with clustering of gephyrin and GABA_A_R at the synapses by a brief application of NMDA (3 min, 20 ${\upmu }$M) [42]. Two-way ANOVA followed by Bonferroni’s post hoc test revealed that NMDA induced significant enhancements of gephyrin (*p* < 0.01) and GABA_A_R β3 subunit (*p* < 0.001) levels in the synaptoneurosomal fraction from the amygdala of the saline-exposed offspring, but not in both of the F1 and F2 generations of the VPA-induced offspring. Moreover, significant effects of generation and NMDA treatment, and significant interaction between generation and treatment were found on synaptic levels of gephyrin and GABA_A_R β3 subunit (gephyrin: generation effect, *F*_(2,36)_ = 83.93, *p* < 0.0001; treatment effect, *F*_(1,36)_ = 16.23, *p* = 0.0003; interaction, *F*_(2,36)_ = 3.637, *p* = 0.0364. GABA_A_R β3 subunit: generation effect, *F*_(2,48)_ = 61.96, *p* < 0.0001; treatment effect, *F*_(1,48)_ = 9.658, *p* = 0.0032; interaction, *F*_(2,48)_ = 6.636, *p* = 0.0029; Fig. 4a–d). Likewise, two-way ANOVA followed by Bonferroni’s post hoc test revealed an increase in mIPSC amplitude (*p* < 0.001) but not frequency after NMDA application in saline-exposed offspring, whereas no significant difference were measured in both generations of the VPA-induced offspring. Furthermore, significant effects of generation and NMDA treatment, and significant interaction between generation and treatment were found on mIPSC amplitude rather than frequency (mIPSC amplitude: generation effect, *F*_(2,50)_ = 100.7, *p* < 0.0001; treatment effect, *F*_(1,50)_ = 10.29, *p* = 0.0023; interaction, *F*_(2,50)_ = 14.56, *p* < 0.0001. mIPSC frequency: generation effect, *F*_(2,50)_ = 48.36, *p* < 0.0001; treatment effect, *F*_(1,50)_ = 0.1254, *p* = 0.7248; interaction, *F*_(2,50)_ = 0.09772, *p* = 0.9071; Fig. 4e–k). Collectively, these data revealed that NMDA-induced gephyrin and GABA_A_R enhancements at the synapses was defective in two generations of the VPA-induced offspring; moreover, the significant generation effects suggest differential GABAergic potentiation in response to NMDA treatment between generations.

### Enhanced glutamatergic modifications across generations of the VPA-induced offspring

**Fig. 5:**
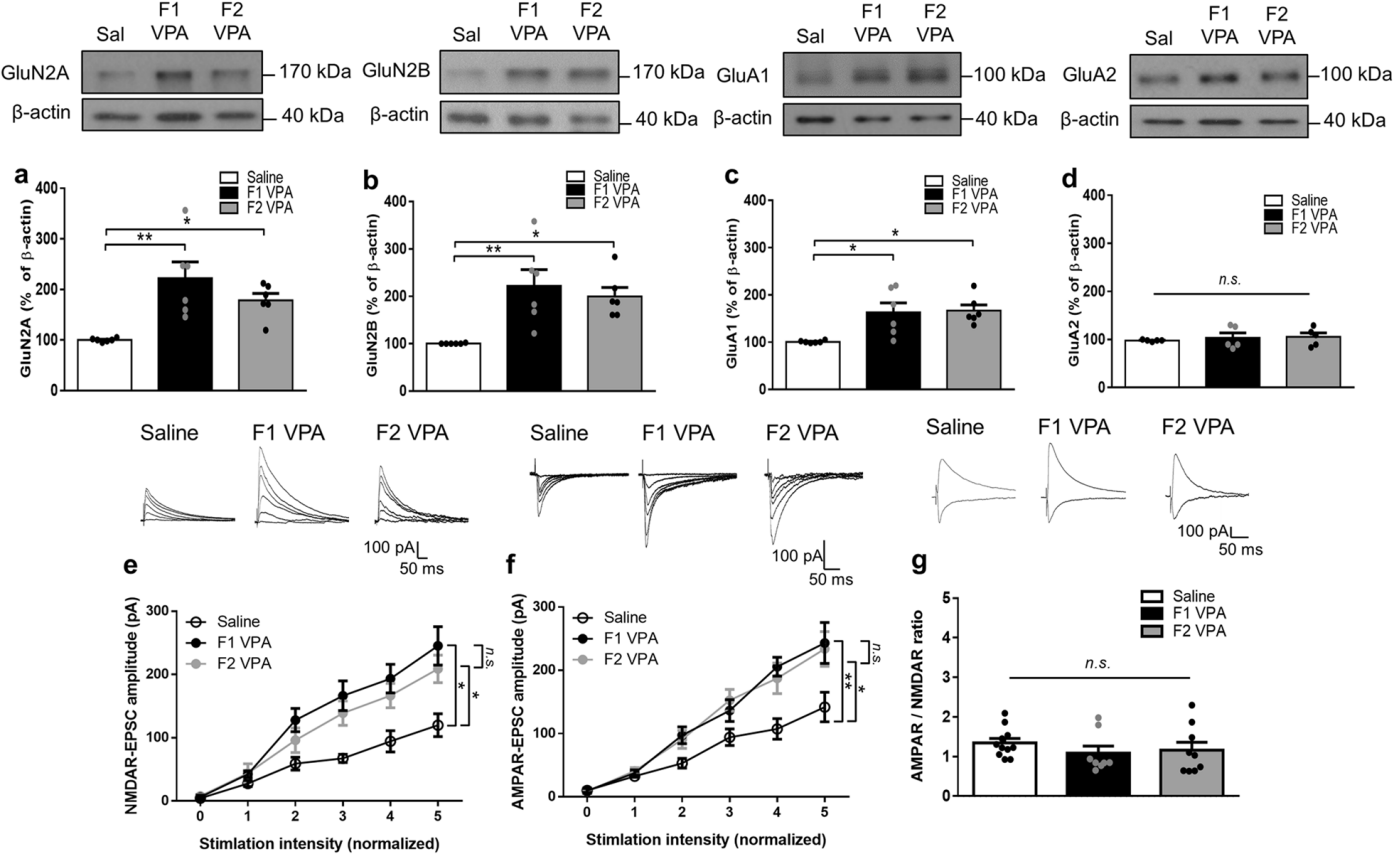
Increased glutamatergic modifications across generations of the VPA-induced offspring. **a–d** Representative western blot profile and the protein levels of GluN2A (**a**), GluN2B (**b**), GluA1 (**c**), and GluA2 (**d**) in synaptoneurosomes from the amygdala. *n* = 5–6 rats from three or four litters for each condition. **e**,** f** Top, Representative EPSC traces at different stimuli. Scale bars, 100 pA, 50 ms. Bottom, input–output curves of NMDAR-EPSC (**e**) and AMPAR-EPSC (**f**) in response to a series of increasing stimulus intensities in the BLA pyramidal neurons. *n* = 6–7 cells from 4–5 rats from three or four litters for each condition. **g** Representative traces and bar graph show the AMPAR- to NMDAR-EPSC ratio measured from each group. Scale bars, 100 pA, 50 ms. *n* = 8–11 cells from 4–5 rats from three or four litters for each condition. Data are presented as mean ± SEM; *p < 0.05, **p < 0.01 vs. indicated control; one-way ANOVA or two-way rmANOVA with Bonferroni post-hoc

In addition to the dampened inhibitory tone observed in the present study, we also examined the alterations in the excitatory glutamatergic system in the VPA model. To accomplish this, we measured the expression profile and synaptic transmission performed by ionotropic glutamate receptors, NMDARs and AMPARs across two generations of the VPA-induced offspring. We observed that the protein levels of GluN2A, GluN2B, and GluA1, but not GluA2 subunits, were significantly increased in synaptoneurosomal tissue from the amygdala of both generations of the VPA-induced offspring (GluN2A: *F*_(2,15)_ = 9.446, *p* = 0.0022; GluN2B: *F*_(2,15)_ = 8.312, *p* = 0.0037; GluA1: *F*_(2,15)_ = 7.532, *p* = 0.0054; GluA2: *F*_(2,12)_ = 0.2646, *p* = 0.7719; Fig. 5a–d). The whole-cell patch clamp measurements revealed that the input–output curves of NMDAR-EPSC were significantly enhanced in the amygdala of both generations of the VPA-induced offspring, and no significant difference was found between the F1 and F2 generations of the VPA-induced offspring (Saline vs. F1 VPA, interaction: *F*_(10,96)_ = 2.241, *p* = 0.0214; Saline vs. F2 VPA, interaction: *F*_(5,66)_ = 2.528, *p* = 0.0373; F1 VPA vs. F2 VPA, interaction: *F*_(5,66)_ = 0.4218, *p* = 0.8319; Fig. 5e). Likewise, the input-output relationships of AMPAR-EPSC were significantly elevated in both generations of the VPA-induced offspring, and no significant difference was found between the F1 and F2 generations of the VPA-induced offspring (Saline vs. F1 VPA, interaction: *F*_(5,60)_ = 4.065, *p* = 0.0030; Saline vs. F2 VPA, interaction: *F*_(5,66)_ = 2.758, *p* = 0.0253; F1 VPA vs. F2 VPA, interaction: *F*_(5,66)_ = 0.2272, *p* = 0.9495; Fig. 5f). The AMPAR/NMDAR ratio remained unchanged in the amygdala of both generations of the VPA-induced offspring compared with the saline-exposed offspring (*F*_(2,25)_ = 0.7145, *p* = 0.4992; Fig. 5g). These results indicated enhanced expression and function of ionotropic glutamate receptors in the amygdala of both F1 and F2 generations of the VPA-induced offspring without difference.

## Discussion

In this study, we provide novel evidence about the E/I synaptic impairments in the amygdala from two generations of the VPA-induced offspring. Importantly, we characterize the GABAergic deteriorations across generations of the VPA model, which specifically, include the greater reductions in GABA_A_R and gephyrin levels, and the loss of GABA_A_R-gephyrin interaction compounding with defective NMDA-induced enhancements of gephyrin and GABA_A_R at the synapses in the F2 generation. These results suggest a potential therapeutic role of GABAergic system in the generational pathophysiology of ASD.

The VPA model is a widely used animal model of ASD owing to its clinical relevance, for a 7–10 fold increased relative risk for ASD [45, 46], and validity [47], for similar behavioral and physiological features with patients. Other than genetic models, the VPA model provides a global aspect of idiopathic ASD, with both environmental and epigenetic origins contributing to 85–95% of ASD cases [48]. Furthermore, the histone deacetylase (HDAC) inhibitory role of VPA may offer a straightforward way to identify epigenetic patterns across ASD generations. These aspects suggest that the VPA model is a valid tool with its unique advantage of studying the cross-generational effects of ASD. The present and previous studies have shown that behavioral and cerebellar abnormalities of the F1 generation in VPA-exposed offspring were transferred to the F2 or F3 generations [31, 49]. This is consistent with previous research revealing that environmental exposure influences not only the exposed individual, but also future generations [10, 50–55]. The proposed mechanism underlying these cross-generational effects is that epigenetic modifications in parental somatic and germ cells may be imprinted onto the offspring [56]. Recent human and animal studies have discovered various mechanisms underlying epigenetic inheritance of neurodevelopmental disorders, such as DNA methylation, histone modifications, and small non-coding RNAs [57, 58]. Alterations in the transcriptome profiles from different generations with neurodevelopmental disorders have been implicated as the consequence of cross-generational epigenetic regulation [13, 30]. Accordingly, it is reasonable to conclude that epigenetic regulation is implicated in the cross-generational effects observed in the VPA-induced ASD model of the present study.

Transient histone hyperacetylation following VPA exposure has been found in the embryonic brain of offspring with ASD [59], further leading to alterations in downstream ASD-related genes [47, 60]. Suppression of genes responsible for the development of GABAergic neurons was found through HDAC inhibition by VPA [61–63]. These findings suggest that the HDAC inhibitory function of VPA may contribute to the decreased GABAergic function in ASD offspring. Dysregulation of GABA_A_R-mediated neuronal inhibition is widely considered as the major etiology of a variety of neurodevelopmental disorders [32, 64]. Clinically, approximately 20–35% reductions in surface GABA_A_R expressions were found in critical brain regions of patients with ASD [28]. In addition, modifications of the GABA_A_R β3 subunit have been emphasized for their direct contribution to the pathophysiology of animal models of ASD [36, 37]. In line with the implications of GABA_A_R dysregulations in ASD patients and animals, the present study provides first demonstration about the reductions in synaptic GABA_A_R β3 subunit levels and GABAergic transmission from the F1 generation up to the F2 generation in ASD offspring induced by prenatal VPA exposure. These observations suggest GABAergic modulations as a general pathogenic mechanism across generations of animal model of ASD.

One of the most critical findings in the present study is the reductions of synaptic GABA_A_R levels and the amplitude of mIPSCs in two generations of the VPA-induced offspring with deteriorations in the F2 generation than the F1 generation. These findings indicate decreased GABAergic postsynaptic function across two generations of the VPA-induced offspring, and the dysfunctions is worsened in the F2 generation. In contrast, the present and previous studies revealed similar reductions of mIPSC frequency and inhibitory presynaptic marker expression [31] in both the F1 and F2 generations of VPA-induced offspring. These observations show that GABAergic deterioration in the F2 generation of our ASD model specifically occurs in the postsynaptic region, rather than in the presynaptic region.

Considering the principal role of gephyrin in stabilizing of GABA_A_R at the synaptic region [65], we found that the synaptic levels of gephyrin were eliminated in two generations of the VPA-induced offspring. There is lower gephyrin expression in the F2 generation than in the F1 generation. That is, the expression profile of synaptic gephyrin is parallel to synaptic GABA_A_R in two generations of individuals with ASD. Moreover, our results further demonstrated a greater loss of gephyrin and GABA_A_R association in the F2 generation than in the F1 generation. This suggests a novel physiological role for gephyrin-associated GABA_A_R β3 subunit in the cross-generational effects of ASD pathophysiology under VPA exposure. Together with previous mechanistic understandings [66–68], our present identification of the gephyrin involvement in synaptic GABA_A_R destabilization and the reduced amplitude of mIPSCs in the F2 generation of our ASD model supporting a great impact of dysregulated gephyrin on the altered postsynaptic GABA_A_R levels in cross-generational ASD pathophysiology.

In the present study, briefly applying NMDA enhances of both GABA_A_R and gephyrin in the postsynaptic region. Besides, NMDA treatment could also trigger AMPAR internalization at the excitatory synapses resulting in the depression of glutamatergic synapses [69]. Aberrant NMDA-induced AMPAR endocytosis in the VPA-induced ASD animals has been found in our previous study [41]. Together with these evidence, the impairments of both GABA_A_R exocytosis and AMPAR endocytosis triggered via the moderate NMDAR activation protocol were well-demonstrated in the present ASD model. On the other hand, phosphorylation of target proteins, including the GABA_A_R β3 subunit, via Ca^2+^/calmodulin protein kinase II (CaMKII) after applying NMDA stimulates the rapid insertion of GABA_A_R at the cell surface with enhanced GABAergic currents [42, 69–71]. Accordingly, we potentiated synaptic GABA_A_R expression and transmission, accompanied by increased gephyrin levels in the control group after NMDA treatment. In contrast, aberrant responses to NMDA treatment were found in the GABAergic synapses of two generations of VPA-induced offspring, with significant generational effects. These results indicate a differential GABA_A_R insertion triggered by NMDA across generations of the present ASD animals. Even though GABA_A_R trafficking into the cell surface is the proposed mechanism, we still cannot rule out the possible enhancements of gephyrin and GABA_A_R association under NMDA treatment from our and other findings. Increased gephyrin levels at the synapses are present alongside increased GABA_A_R levels. Enhanced GABAergic transmission was also identified following NMDA treatment in our and other studies [42]. Moreover, manipulation of gephyrin by gene knockout, antisense approaches, and identification of post-translational modifications at specific gephyrin residues highlight the critical role of gephyrin in synaptic GABA_A_R clustering [72–75]. These findings suggest that not only the insertion of GABA_A_R, but also enhancement of gephyrin and GABA_A_R association contributes to the NMDA-enhanced effects on GABAergic synapses. The present findings in either the gephyrin-associated GABA_A_R β3 subunit, or the NMDA-induced effects on GABAergic synapses is sufficient to explain the dependence of gephyrin on GABAergic deteriorations at the postsynaptic region across generations of VPA-induced animals with ASD. The functional interplay of gephyrin with synaptic proteins, such as neurexins and neuroligins, implicated in neurodevelopmental diseases has long been discovered [76, 77]. Copy number variations and exonic deletions in the *GPHN* gene have been identified in individuals with ASD [78, 79], uncovering strong human genetic evidence for the involvement of gephyrin in the pathogenesis of ASD. The present study uncovered a previously unknown pathogenic role of gephyrin compromised in the generational effects on GABA_A_R expression and function among the pathophysiology of ASD.

A strong linkage between glutamatergic dysfunctions in the brain and behavioral abnormalities in the ASD animals was well-identified in previous studies [20, 21, 80, 81]. We revealed the enhancements of NMDAR and AMPAR expressions and currents in the amygdala from both F1 and F2 generations of the VPA-induced offspring. Increased synaptic levels of GluN2A, GluN2B, and GluA1, rather than GluA2, were observed. This is consistent with previous findings that GluA2-containing AMPAR endocytosis is abnormal in the VPA-exposed offspring, and the baseline synaptic GluA2 levels remain unchanged [41]. While the AMPAR/NMDAR ratio is a strong index of the synaptic state, particularly contributed by postsynaptic AMPARs and NMDARs, no significant difference was found in the two generations of VPA-induced offspring. Together with previous findings [20, 82], one possible explanation may be the similar enhancement of both NMDAR- and AMPAR-mediated currents in the VPA-induced offspring. Previous research showed a significantly altered rectification of AMPAR-mediated currents with an unaltered AMPAR/NMDAR ratio [83], suggesting further examinations to elucidate the detailed contributions of each subunit. In a brief summary, these findings suggest that the contribution of glutamatergic tone cannot be ruled out in the cross-generational effects of ASD. More importantly, through the discovery of deteriorations in GABAergic system rather than in glutamatergic system in the F2 generation than the F1 generation of the ASD offspring, great attentions on the role of GABAergic modulation in the cross-generational effects of ASD pathogenesis must be paid.

In the present study, increased glutamatergic receptor expressions and functions, and declined GABAergic receptor expressions and functions at the synaptic levels were demonstrated in the amygdala from two generations of the VPA-induced offspring. In consistency, a prior study on the prefrontal cortex (PFC) illustrated enhanced glutamate-related proteins and reduced GABA-related proteins up to the F2 generation of the VPA-exposed offspring [31]. Given the central role of both the amygdala and PFC in social/emotional brain circuits [20, 84, 85], these observations support E/I imbalance as the hallmark feature in the cross-generational effects of ASD causing ASD-like behavioral phenotypes in the subsequent generations.

In addition to the present findings on the cross-generational effects of VPA-induced ASD in animals, a study on human recently reported the adverse neurodevelopmental outcomes of VPA, including malformations and neurodevelopmental disorders, diagnosed from about 53% of F2 generations [86]. Clinical studies have recently suggested that multigenerational risks increase the prevalence of ASD under accumulated epigenetic alterations reflecting past environmental exposure levels and life history [87–90]. Previous evidence has shown differing or worsening behavioral performance in the F2 generation than in the F1 generation, with different transcriptomic changes in models exposed to environmental insults [10, 13]. This indicates that accumulation of epigenetic modifications may induce different pathological conditions across generations of ASD. The present demonstration of profound GABAergic phenotypes in the F2 generation compared to the F1 generation of the VPA model further provides a novel biomarker for the multigenerational effects of ASD. In support of this, a preclinical study showed the beneficial effects of prenatal GABA_B_R agonist treatment on the F2 generation of the VPA model [91]. These findings suggest that dysfunction in GABAergic system is a critical pathological mechanism that provides an important direction for the therapeutic strategy of cross-generational effects on ASD.

## Conclusions

The present study is the first supportive demonstration of E/I disturbances as the central hypothesis in the cross-generational pathogenesis of F2 generation of the ASD animals. More intriguingly, significant deteriorations were found in the GABAergic system rather than the glutamatergic system of the F2 generation than the F1 generation providing novel perspectives on the cross-generational effects of ASD pathophysiology on inhibitory synaptic dysfunctions. Mechanistically, our findings on either GABA_A_R-gephyrin interaction at the synaptic level or NMDA-induced enhancements of gephyrin and GABA_A_R offer valuable insight into the novel regulatory role of gephyrin in both GABA_A_R expression and function across generations of the ASD offspring. Overall, these observations suggest that targeting the GABAergic system may be a viable therapeutic approach toward correcting the generational pathophysiology of ASD which is also predictive for neurodevelopmental disorders with at-risk early-life experience, or parental transmission into subsequent generations.

## Supplementary Information

**Additional file 1: Figure S1.** Experimental design for measuring the autism-like phenotypes across two generations of the VPA-induced offspring. (a) Strategies of producing the F1 and F2 generations of the VPA-induced offspring. (b) Timeline of experimental procedures. Five-day behavioral assays were conducted from PND28 to PND32, including three-chamber social test (PND28), open field test (PND29), elevated plus maze test (PND30), marble burying test (PND31) and forced swim test (PND32). Western blotting and electrophysiological recordings were performed soon after the behavioral assays. E, embryonic day; PND, postnatal day

## Data Availability

All data generated or analysed during this study are included in this published article.

## Abbreviations

AMPAR: α-Amino-3-hydroxy-5-methyl-4-isoxazolepropionic acid receptor
ASD: Autism spectrum disorder
BLA: Basolateral amygdala
E: Embryonic day
E/I: Excitatory/inhibitory
EPSC: Excitatory postsynaptic potential
GABA_A_R: γ-Aminobutyric acid type A receptor
GluA1: AMPAR receptor subunit type 1
GluA2: AMPAR receptor subunit type 2
mIPSC: Miniature inhibitory postsynaptic current
NMDAR: *N*-methyl-d-aspartate receptor
GluN2A: NMDA receptor subunit 2 A
GluN2B: NMDA receptor subunit 2B
PND: Postnatal day
VPA: Valproate
