# Correction: Generational synaptic functions of GABA_A_ receptor β3 subunit deteriorations in an animal model of social deficit

**DOI:** 10.1186/s12929-023-00904-8

**Published:** 2023-02-16

**Authors:** Ming Chia Chu, Han Fang Wu, Chi Wei Lee, Yueh Jung Chung, Hsiang Chi, Po See Chen, Hui Ching Lin

**Affiliations:** 1 Department and Institute of Physiology, School of Medicine, National Yang Ming Chiao Tung University, Taipei, 112 Taiwan; 2 Ph.D. Program for Neural Regenerative Medicine, College of Medical Science and Technology, Taipei Medical University and National Health Research Institutes, Taipei, 110 Taiwan; 3 Department of Psychiatry, National Cheng Kung University Hospital, College of Medicine, National Cheng Kung University, Tainan, 704 Taiwan; 4 Institute of Behavioral Medicine, College of Medicine, National Cheng Kung University, Tainan, 704 Taiwan; 5 Brain Research Center, National Yang Ming Chiao Tung University, Taipei, 112 Taiwan

**Correction: Journal of Biomedical Science (2022) 29:51** **https://doi.org/10.1186/s12929-022-00835-w**

Following publication of the original article [1], it was found the affiliations 1 and affiliation 3 are incorrect for the authors, the correct affiliation 1 and affiliation 3 should be:

^1^ Department and Institute of Physiology, School of Medicine, National Yang Ming Chiao Tung University, Taipei, 112, Taiwan.

^3^ Department of Psychiatry, National Cheng Kung University Hospital, College of Medicine, National Cheng Kung University, Tainan, 704, Taiwan;

The original publication has been corrected.
