# Supplementary material for: Generational synaptic functions of GABAA receptor β3 subunit deteriorations in an animal model of social deficit
Source: J Biomed Sci. 2022 Jul 11;29:51. doi: 10.1186/s12929-022-00835-w (PMC9277936; doi:10.1186/s12929-022-00835-w)
Supplement: Supplementary file 1 — Additional file 1: Figure S1. Experimental design for measuring the autism-like phenotypes across two generations of the VPA-induced offspring. (a) Strategies of producing the F1 and F2 generations of the VPA-induced offspring. (b) Timeline of experimental procedures. Five-day behavioral assays were conducted from PND28 to PND32, including three-chamber social test (PND28), open field test (PND29), elevated plus maze test (PND30), marble burying test (PND31) and forced swim test (PND32). Western blotting and electrophysiological recordings were performed soon after the behavioral assays. E, embryonic day; PND, postnatal day [file 12929_2022_835_MOESM1_ESM.docx]

**Additional File 1**

**Generational synaptic functions of GABA_A_ receptor β3 subunit deteriorations in an animal model of social deficit**

Ming-Chia Chu^1^, Han-Fang Wu^1^, Chi-Wei Lee^1^, Yueh-Jung Chung^1^, Hsiang Chi^1^, Po See Chen^3, 4, *^ and Hui-Ching Lin^1, 2, 5, *^

**Figure S1**

**
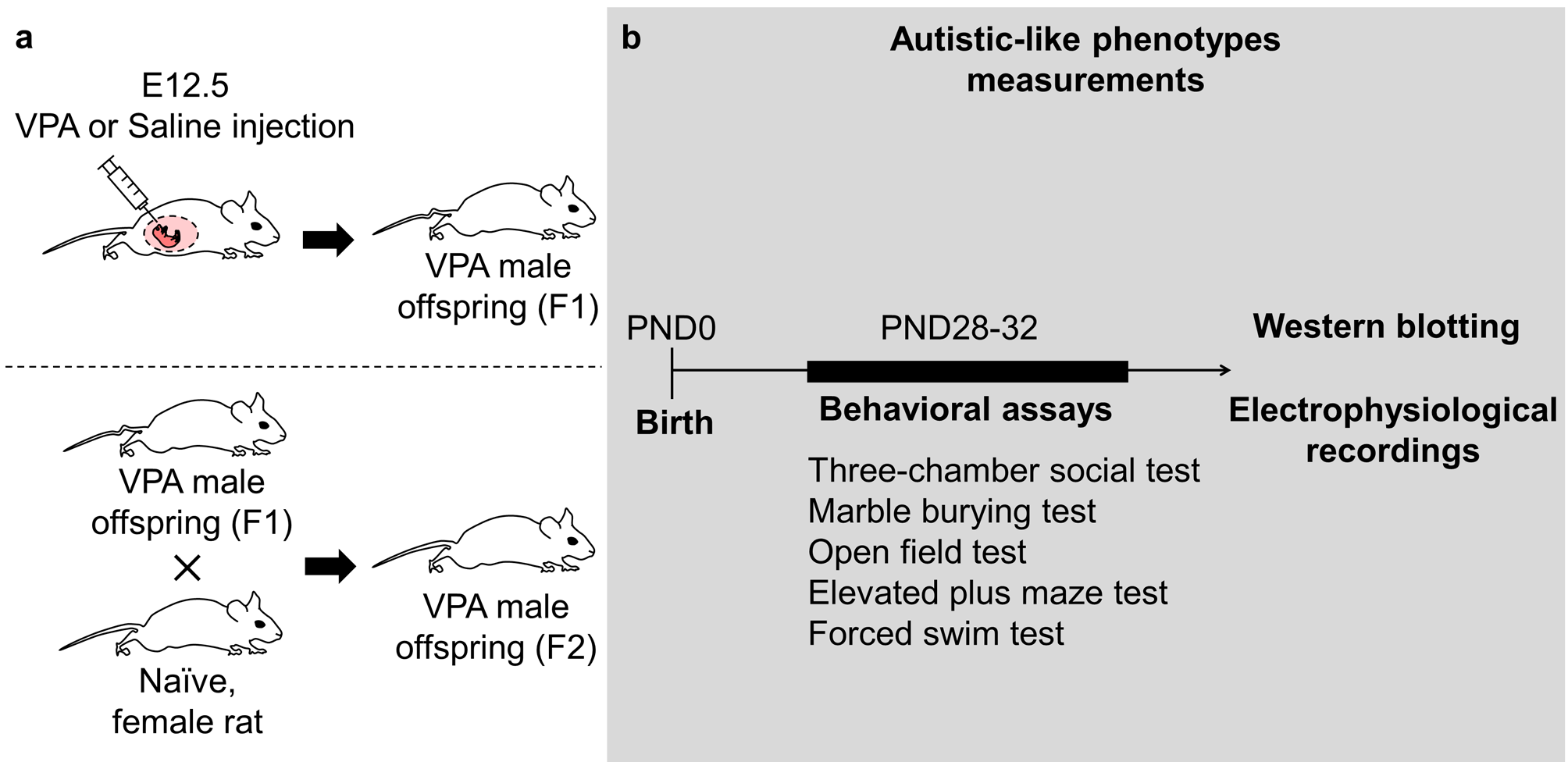
**

**Fig. S1** Experimental design for measuring the autism-like phenotypes across two generations of the VPA-induced offspring. (**a**) Strategies of producing the F1 and F2 generations of the VPA-induced offspring. (**b**) Timeline of experimental procedures. Five-day behavioral assays were conducted from PND28 to PND32, including three-chamber social test (PND28), open field test (PND29), elevated plus maze test (PND30), marble burying test (PND31) and forced swim test (PND32). Western blotting and electrophysiological recordings were performed soon after the behavioral assays. E, embryonic day; PND, postnatal day.
